# Reliability and validity of the Chinese version of the trauma-specific frailty index (TSFI) for geriatric trauma patients

**DOI:** 10.1186/s12877-023-04243-z

**Published:** 2023-10-02

**Authors:** Ding-Xi Bai, Yun Liang, Chen‐Xi Wu, Chao-ming Hou, Jing Gao

**Affiliations:** Chengdu University of Traditional Chinese Medicine, 1166 Liutai Avenue, Chengdu, 611137 China

**Keywords:** Trauma, Frailty, Trauma-specific Frailty Index, Reliability, Validity

## Abstract

**Background:**

Pre-traumatic frailty in geriatric trauma patients has caught attention from emergency medical workers and the assessment of it thus become one of the important aspects of risk management. Several tools are available to identify frailty, but limited tools have been validated for geriatric trauma patients in China to assess pre-traumatic frailty.The aim of this study is to translate the Trauma-Specific Frailty Index(TSFI) into Chinese, and to evaluate the reliability and validity of the translated version in geriatric trauma patients.

**Methods:**

A cross-sectional study was conducted. The TSFI was translated with using the Brislin model, that included forward and backward translation. A total of 184 geriatric trauma patients were recruited by a convenience sampling between October and December 2020 in Hospital of Chengdu University of Traditional Chinese Medicine, Sichuan. Using reliability or internal consistency tests assessed with Cronbach’s alpha coefficient, split-half reliability and test-retest reliability. Content validity and construct validity analysis were both performed. Sensitivity, specificity and maximum Youden index(YI) were used to determine the optimal cut-off value. The screening performance was examined by Kappa value.

**Results:**

The total study population included 184 subjects, of which 8 participants were excluded, resulting in a study sample size of 176 elderly trauma patients (the completion rate was 95.7%). The Chinese version of Trauma-Specific Frailty Index(C-TSFI) have 15 items with 5 dimensions. Cronbach’s alpha coefficient of the C-TSFI was 0.861, Cronbach’s alpha coefficient of dimensions ranged from 0.837 to 0.875, the split-half reliability of the C-TSFI were 0.894 and 0.880 respectively, test-retest reliability ranged from 0.692 to 0.862. The correlation coefficient between items and the C-TSFI ranged from 0.439 to 0.761. The content validity index for items (I-CVI) of the C-TSFI scale was 0.86~1.00, and the scale of content validity index (S-CVI) was 0.93. The area under curve (AUC) of the C-TSFI was 0.932 (95%CI 0.904–0.96, *P* < 0.05), the maximum YI was 0.725, the sensitivity was 80.2%, the specificity was 92.3%, and the critical value was 0.31. Kappa value was 0.682 (*P* < 0.05).

**Conclusions:**

The Chinese version of TSFI could be used as a general assessment tool in geriatric trauma patients, and both its reliability and validity have been demonstrated.

**Supplementary Information:**

The online version contains supplementary material available at 10.1186/s12877-023-04243-z.

## Introduction

Trauma, a global public health problem [1], refers to the direct or indirect destruction of the structural integrity of human tissues under the action of external mechanical factors. It has the characteristics of suddenness, high incidence, high mortality and high disability. Nowadays the whole world is confronted with the problem of aging population and China is no exception, the incidence of geriatric trauma is increasing, becoming the fifth leading cause of abnormal deaths in the elderly [2]. Compared with the young people, the old are more likely to have serious adverse outcomes in face of trauma because of the deterioration of their body function [3]. Even with the same injury severity, elderly trauma patients in the emergency department have a higher mortality rate than younger trauma patients [4–8]. This lead to negative clinical consequences, such as the increase of hospitalization expense and extension of hospitalization time, thus reducing the quality of life of the elderly [9–11]. It is reported that about 49% of geriatric trauma patients in emergengcy department have not received effective management, due to risk factors not be identified availably [12]. So it is urgent to have tools that can effectively assess the risk factors, and screen out the high-risk groups.

In recent decades, more and more studies have shown that frailty is closely related to the adverse outcomes of elderly trauma patients. It is very important for the post-traumatic outcome of the elderly whether they have frailty before the trauma. And by evaluating frailty, whose predictive power of adverse outcomes is superior than that of age and trauma severity scores for elderly trauma patients [13, 14]. The American Association for Surgery of Trauma (AAST) recommends identifying the patient’s level of frailty as early as possible in the treatment of geriatric trauma patients [4]. There are more than 70 measurement instruments have been developed to assess frailty [7]. Although Frailty Phenotype [5], Frail Scale [15], Frailty index [16]and Tilburg Frailty Indicator [17] have been widely cited in the literature, these tools some are somewhat simple and lack feasibility, for intstance, grip strength and walking speed are difficult to identify for elderly trauma patients. While Tilburg Frailty Indicator is comlex and time comsuming. Trauma Specific Frailty Index (TSFI), developed in 2014 by American Professor Bellal Joseph, has proved to be convenient, simple, time-saving, easy to operate and has a higher predictive power in clinical application. [13] It can assess eldely trauma patients’ frailty status and predicte the risk of adverse outcomes in emergency department. TSFI, according to report, has become a common risk assessment tool for emergency elderly trauma patients in the field of clinical practice [18].

Taking these aspects into account, together with the fact that in China few validated instruments for assessing geratric trauma patients pre-traumatic frailty status.

Therefore, the study has two objectives: (1) to translate the English version of the TSFI into the Chinese and make necessary adaptaion, to enrich relevant scales to provide a wider range of choices ; (2) to evaluate the reliability and validity of the Chinese version of the TSFI in geriatric trauma patients from Chinese emergency departments.

## Methods

### Design, setting and participants

This is a descriptive cross-sectional study that included 184 participants from the Hospital of Chengdu University of TCM, Sichuan, China between October and December 2020. The sample was selected by means of a convenience method and the participants’ consent has been obtained. According to Kendall’s guidelines [19], Since the sample size should be 5 to 10 times the number of items on the TSFI scale, the variable was 15, at least 150 cases should be included, and considering the 20% sample loss rate, hence a total of 180 patients. This study was actually included 184.

In order to be included and be able to participate in the study, the participants had to meet the following inclusion criteria: (1) be of either sex, aged 60 years or over(in China, 60 years old is the standard to determine the division of the elderly); (2) trauma patients whose integrity of body tissues and organs is destroyed due to various reasons(such as falls, traffic accidents) ; (3) the time of admission after trauma is less than 24 h; (4) voluntarily signing the informed consent form.

The exclusion criteria are: (1) elderly trauma patients with Injury Severity Score(ISS) at 25 or more in the emergency department, (2) not being able to read and having sever hearing loss, (3) disturbance of consciousness, with severe organ failure and end-stage disease, equal to the clinical type of brain death.

The following withdrawal criteria are also considered after inclusion:1) abandoning the study anytime in the process despite having signed of informed consent form, 2) and completion of less than 50% of the scales administered.

The research stages are as follows: First, the TSFI was converted from the English version through forward- and backward- translation procedure into the Chinese version; secondly, the reliability, validity and feasibility of the Chinese version of TSFI were tested by cross-sectional research design (Cronbach’s alpha coefficient, test-retest reliability, split-half reliability, content validity, Construct validity, criterion-related validity and screening performance test, etc.).

### Questionnaires

#### Trauma specific frailty index(TSFI)

The TSFI, which developed by Professor Bellal Joseph of the University of Arizona in the United States in 2014, is specific to assess eldely trauma patients’ frailty status.

The questionnaire includes 15 items in 5 aspects describing comorbidities, social activities, daily activities, nutritional status and health attitudes. The score of each item of the TSFI scale ranges from 0 (representing non-frail state) to 1 (representing severe frail state). Most of the 15 variables included in TSFI are dichotomous variables (yes or no), while other items are of multiple levels. The TSFI score is average score of the 15 items, ranging from 0 to 1. The higher the score, the weaker the body [13].

#### Frail scale

The FRAIL Scale is a frailty assessment scale developed by a team of geriatric experts from the International Working Group on Nutrition, Health and Aging in 2008. It has good reliability and validity in Chinese hospitalized geriatric patients, with Cronbach’s alpha coefficient of 0.826, Kappa coefficient of 0.892, and S-CVI/Ave of 0.98 [20]. The FRAIL Scale includes fatigue, resistance, walking, illness, and weight loss. Each item is scored between o and 1 point, and the total score for the five items is between 0 and 5 points. If 3 or more items are satisfied with full score, frailty can be diagnosed, 1 to 2 items can be diagnosed as pre-frailty, and 0 items can be diagnosed as no frailty [21].

### General Condition Questionnaire

The self-designed General Condition Questionnaire was used to evaluate the demographic data, and the content includes: (a)sex; (b)age; (c)degree of education; (d)marital status; (e)income and expenses; (f)smoking; (g)drinking. Clinical data: (a)cause of injury; (b)location of injury; (c)nature of injury;(d)ISS score.

### Translation process

The research group first contacted the TSFI group in America by E-mail and obtained authorization for the scale. Then the translation of the scale was conducte by using the Brislin model.

The translation procedure was as follows:

#### Step 1 initial translation

Two Chinese native speakers proficient in English translated the original version of TSFI into Mandarin (simplified) Chinese version independently. The first translator was a clinical nurse with master degree from hospital of Chengdu University of TCM who was invited to translate the questionnaire from clinical perspective. The second one was a non-medical translator who was translated the questionnaire by language aspect only.

#### Step 2 synthesis of the translations

The research team held the discussion to review the two Chinese versions of TSFI in comparison with the original English version. Chinese words were carefully chosen from both translations and some modifications were made to construct a single version of the questionnaire finally.

#### Step 3 backward translation

Another two Chinese-English translators who had never seen the original English version were asked to translate the reconciled Mandarin (simplified) Chinese version back into English. The research team held the discussion to compare the original English version with the backward translated English version, and then made further modifications. After that, the modified reconciled Chinese version was once again translated back into English. With several forwards and backwards, the backward-translated English version got as close to the original as possible, and up till now, a pre-investigation Chinese version of TSFI was finally formed.

### Pilot testing and culture adaptation

The pre-investigation Chinese version of TSFI was first tested on 30 selected patients. The necessary modifications were then made based on their suggestions and the Chinese version of TSFI finally came out.

### Data collection

This study followed all principles outlined in the Declaration of Helsinki. Patients were informed about the aims of the study, confidentiality of the data and voluntary participation. All participants signed informed consent. Each participant did the General Condition Questionnaire, TSFI and Frail Scale during hospital admission.

### Statistical analysis

All statistical analyses were conducted via SPSS 21.0. Number (percentage) was used to describe enumeration data, and mean ± standard deviation (SD) was adopted to describe continuous data. Internal consistency reliability for each domain and total of the scale was determined with Cronbach’s α coefficient. According to the literature, Cronbach’s α greater than 0.9 was considered as excellent, 0.8–0.9 as good, 0.7–0.8 as acceptable, 0.6–0.7 as questionable, 0.5–0.6 as poor and lower than 0.5 as unacceptable [22].Split-half analysis was also used to evaluate the reliability of internal consistency.Generally the split-half is greater than or equal to 0.7 based on the literature [23]. The test-retest reliability was examined using the intra-class correlation (ICC), which were evaluated using Pearson coefficient. The ICC value can be determined by reference to the following: poor (less than 0.5), moderate (between 0.5 and 0.75), good (between 0.75 and 0.9) and excellent (greater than 0.90) [24].

Content validity was assessed. The C-TSFI were evaluated by four clinical emergency specialists and three geriatric care specialists who were not involved in the translation process. Each item of the translated questionnaire was rated in a 4-point scale of ‘1 = not relevant’, ‘2 = somewhat relevant’, ‘3 = quite relevant’ and ‘4 = highly relevant’.

To analyse the construct validity of the C-TSFI, correlation tables were used, which were resolved using Pearson’s Correlation Coefficient. Correlations were made between the scores of each item and the total score the of the C-TSFI.

Pearson’s correlation coefficient between the FRAIL scale and the C-TSFI scale was calculated to test the correlation validity of the criterion. The Frailty were screened with scores ≥ 3 on the Frailty scale. The elderly emergency trauma patients were divided into frailty group and non-frailty group. And then by plotting the ROC curve, sensitivity, specificity and maximum Youden index were used to determine the optimal cut-off value of the C-TSFI screening for frailty. In addition, the screening performance of the C-TSFI was investigated by calculating the Kappa value.

## Results

### Descriptive statistics

A total of 184 elderly truama patients were enrolled into the study, 176 questionnaires were effectively recovered, with an effective recovery rate of 95.7%. 8 participants were excluded because they met some withdrawal criteria. The mean age of patients was 72.13years(± 7.85years), 77 males and 99 females. The education of the majority was junior high school or below, accounting for 63.1%(Table 1).

**Table 1:** Characteristics of study participants

Characteristics(n = 176)	[$\bar {x}$± *s*/(*n*)*%*]	Characteristics(n = 176)	[$\bar {x}$± *s*/(*n*)*%*]
Age	72.13 ± 7.85	Surplus	78(44.3%)
Sex		Make both ends meet	71(40.3%)
Male	77(43.8%)	Cannot make ends meet	27(15.3%)
Female	99(56.2%)	Mechanism of injury	
Education		Fall	54(30.7%)
Junior school and below	111(63.1%)	MVC	93(52.8%)
Senior high school	49(27.8%)	Others	29(16.5%)
Bachelor and above	16 (9.1%)	Injury Severity Parameters	
Marital status		Limbs	103(58.5%)
Unmarried	4(2.3%)	Brain	35(19.7%)
Married	148(84.1%)	Others	38(21.8%)
Divorced	5 (2.8%)	Injury Severity Parameters	
Widowed	19(10.8%)	ISS 16	137(77.6%)
Income status		ISS 16	39(22.4%)

### Reliability

This study used internal consistency reliability and test-retest reliability (r) to evaluate the TSFI reliability. Reliability of internal consistency includes Cronbach alpha coefficient and split-half reliability. The Cronbach alpha coefficient of TSFI was 0.861(Table 2). The split-half reliability of C-TSFI was divided into half with 2 patterns. One was according to the odd and even number, another was splited randomly. Guttman Split-Half Coefficient were 0.894 and 0.880 respectively(Table 3). Thirty elderly truama patients in the retest of the C-TSFI by in-person. The overall ICC value for the test-retest reliability was 0.871, and the each dimension of TSFI ranged 0.692 ~ 0.862(Table 2).

**Table 2:** Cronbach alpha coefficient and the test-retest reliability of the C-TSFI

Total C-TSFI/Dimension	Cronbach’α	ICC
Total C-TSFI	0.861	0.871
Comorbidities	0.814	0.862
Daily Activities	0.875	0.761
Health Attitude	0.837	0.827
Function	-	0.692
Nutrition	-	0.813

**Table 3:** The split-half reliability of the C-TSFI

Project	Guttman Split-Half Coefficient	
C-TSFI	split the items by odd and even numbers	split the items randomly
	0.894	0.880

### Validity

It is determined that the I-CVI of the C-TSFI scale is 0.86~1, and the S-CVI of the TSFI is 0.93.

The Pearson correlation coefficient between the score of each item and the total score of the scale ranged from 0.439 to 0.761, and all items were greater than 0.30. The score of each item was positively correlated with the total score, and the difference was statistically significant (*P* < 0.001).(Table 4).

**Fig. 1:**
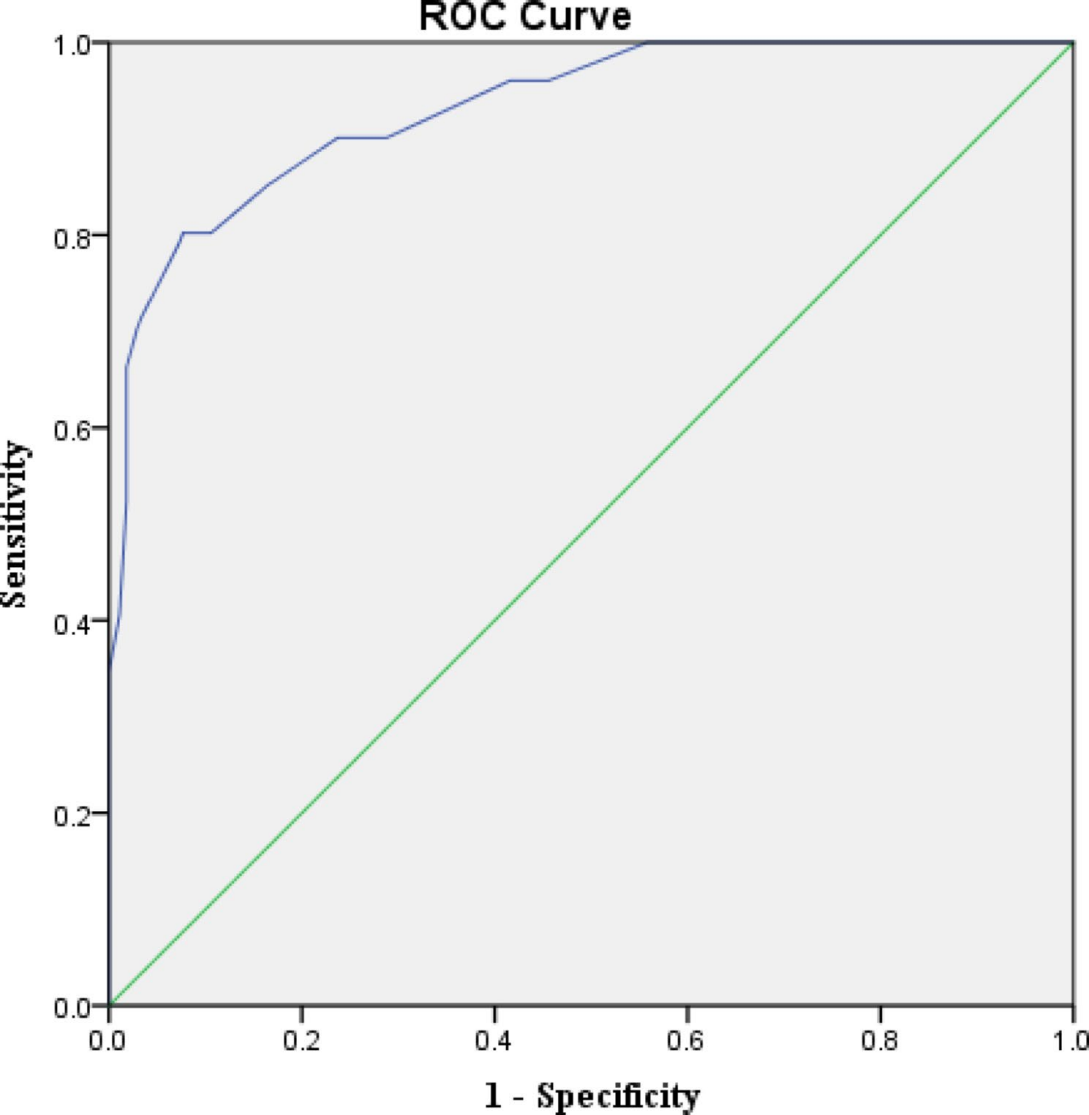
ROC curve of the C-TSFI scale(Frail Scale ≥ 3 as the reference standard)

The C-TSFI was positively correlated with the total score of FRAIL (*r* = 0.820, *P* < 0.05).The Receiver Operating Characteristic (ROC) curve was drawn to calculate the sensitivity, specificity and critical value of the Chinese version of TSFI for frailty to test the screening performance of the scale.The AUC of the C-TSFI scale was 0.932 (95%CI 0.904–0.96, *P* < 0.05), the Youden index was 0.725, the sensitivity was 0.802, the specificity was 0.923, and the best critical value was 0.31 (Table 5; Fig. 1). The Kappa value of FRAIL Scale and the C-TSFI scale was 0.682 (*P* < 0.05).

**Table 4:** Correlation between Chinese version TSFI items and total score

Item	Pearson	*P*
1	0.594	*P* < 0.001
2	0.627	*P* < 0.001
3	0.656	*P* < 0.001
4	0.700	*P* < 0.001
5	0.761	*P* < 0.001
6	0.703	*P* < 0.001
7	0.605	*P* < 0.001
8	0.737	*P* < 0.001
9	0.716	*P* < 0.001
10	0.672	*P* < 0.001
11	0.609	*P* < 0.001
12	0.510	*P* < 0.001
13	0.582	*P* < 0.001
14	0.440	*P* < 0.001
15	0.439	*P* < 0.001

**Table 5:** Sensitivity, specificity and Youden’s index of the Chinese version TSFI scale based Frail Scale ≥ 3 as the reference standard

Chinese version of TSFI score	Sensitivity	Specificity	Youden Index
0.01	1	0.051	0.051
0.025	1	0.069	0.069
0.05	1	0.139	0.139
0.075	1	0.215	0.215
0.09	1	0.318	0.318
0.11	1	0.442	0.442
0.125	0.96	0.544	0.504
0.14	0.96	0.584	0.544
0.16	0.901	0.712	0.613
0.175	0.901	0.734	0.635
0.19	0.901	0.748	0.649
0.21	0.901	0.763	0.664
0.225	0.881	0.792	0.673
0.25	0.851	0.836	0.687
0.275	0.802	0.894	0.696
0.29	0.802	0.901	0.703
**0.31**	**0.802**	**0.923**	**0.725**
0.325	0.792	0.927	0.719
0.34 ~ 0.885	0.713 ~ 0.03	0.967 ~ 1	0.68 ~ 0.03

## Discussion

The incidence of trauma is gradually increasing with the problem of aging globally. And there is a high risk of complications and adverse outcomes [8] due to the suddenness of trauma, the complexity of injury factors and clinical symptoms, and the susceptibility to interference from other factors during the treatment process. There is increasing recognition of the benefit of early identification of frailty to predict outcome or guide resource use in older emergency surgery and trauma patients [25, 26]. We still found that a lack of consensus of how and when frailty should be identified in trauma patients [27]. The British Geriatric Society makes recommendation that frailty assessment occurs across all healthcare setting and in patients with different clinical conditions but do not recommend a specific tool for use in major trauma [28].Therefore, it is important to conduct a comprehensive assessment and predicting the risk in geriatric adult trauma patients by using appropriate tools. Trauma Specific Frailty Index(TSFI) [13], derived from the 50-variable modified Rockwood frailty index is more suitable for implementation in acute trauma situations [14]. As a specific questionnaire based on the deficit accumulation model of frailty, TSFI consists of five main domains: comorbidities, daily activities, health attitude, function, and nutrition with15 variables. Compared with other commonly used clinical frailty assessment tools, TSFI has the advantages of convenience, higher efficiency and predictive power to adapt to the busy and time-sensitive clinical situation in the emergency department [18].

The TSFI was translated with using the Brislin model and achieved satisfying psychometric properities. In addition, this study is the first time to be tested against the FRAIL in China, which was as the reference standard. Finally, we find the TSFI scale and the FRAIL Scale in this study had good consistency within their respective screening thresholds. The TSFI had excellent accuracy and 0.31 could be served as critical value.

To emphasize particularly, In China, Zhao’s group have devolopped the geriatric trauma frailty index(GTFI) for elderly trauma patients based on electronic hospital records, and has been proven to be useful in China [29]. Both of them are convenient and fast to calculate. Compared with the Chinese version of the TSFI(C-TSFI), Zhao indicated that the GTFI can use the routine data of a hospital’s electronic medical records system and it can eliminate the need to manually calculate the score.Their research further pointed out the GTFI had the general consistency with the C-TSFI score [29, 30].

Even so, we consider it is essential to introduce the TSFI in China. Firstly, GTFI is constructed based on the patients’ ICD-10 diagnostic code, While the TSFI based on multi-dimensional health status. Not hard to find, when we use the GTFI, more attention to obvious trauma and chronic diseases, while ignoring some defects in psychological and social adaptation [29, 30]. The TSFI includes these areas, more comprehensive than that. Secondly, the GTFI may not widely promoted and applied because of some electronic devices, software and high cost, while the TSFI is more convenience and free for use. In China, The development of economic conditions in different regions is not consistent. In comparison, the C-TSFI has more greater advantage to apply and promote.

This study is the first to explore the screening value of the C-TSFI and determine critical value. We investigated the correlation with the reference standard, The C-TSFI was positively correlated with the total score of the FRAIL. As we kown, There is no gold standard for the assessment of frailty. The FRAIL as the reference standard in our study based on International clinical practice guidelines [31].Finally, The results of the study showed that the AUC of the C-TSFI scale was 0.932, indicating a high screening value. The maximum Youden index of the C-TSFI was 0.725, the sensitivity was 80.2%, the specificity was 92.3%, and the critical value was 0.31. The critical value was higher than original scale critical value [13].There was substantial agreement between the C-TSFI scale defined frailty and the FRAIL Scale defined frailty(Kappa 0.682, *P* < 0.05). The result was inconsistent with Heather Jarman’s group research, they reported that, compared with the CFS (Clinical Frailty Scale) and PRISMA7 (Program of Research to Integrate Services for the Maintenance of Autonomy 7), the TSFI showed the slightest agreement with geriatrician assessment of frailty [32]. Such result might be related to the inconsistency of the reference standards selected. Further studies are needed to confirm this.

## Conclusions and Limitations

The Chinese version of TSFI is a specific scale dedicated to the assessment of geriatric adult trauma patients. It has good reliability and validity.

However, the sample size included in this study is relatively small, and the survey scope is limited to Chengdu area of Sichuan, China, which may affects the universality of the results. Because, the environment, cultural customs, health cognition and psychological characteristics are diverse in different regions in China.The next step should be to expand the range of sample sources and further verify the Chinese version of TSFI, which can ultimately provide a scientific reference standard for early intervention of traumatic geriatric patients.

### Electronic supplementary material

Below is the link to the electronic supplementary material.

Supplementary Material 1

## Data Availability

The datasets used and/or analyzed during the study are available from the corresponding author on reasonable request.

## Abbreviations

TSFI: Trauma-Specific Frailty Index
C-TSFI: the Chinese version of Trauma-Specific Frailty Index
AAST: the American Association for Surgery of Trauma
ISS: Injury Severity Score
I-CVI: Content validity index for items
S-CVI: Scale of content validity index
YI: Youden Index
AUC: Area Under Curve
ICC: Intra-Class Correlation
ROC: Receiver Operating Characteristic
GTFI: the geriatric trauma frailty index
CFS: Clinical Frailty Scale
PRISMA7: Program of Research to Integrate Services for the Maintenance of Autonomy 7
