# Supplementary material for: Reliability and validity of the Chinese version of the trauma-specific frailty index (TSFI) for geriatric trauma patients
Source: BMC Geriatr. 2023 Oct 2;23:617. doi: 10.1186/s12877-023-04243-z (PMC10546729; doi:10.1186/s12877-023-04243-z)
Supplement: Supplementary file 1 — Supplementary Material 1 [file 12877_2023_4243_MOESM1_ESM.docx]

| *Appendix1 The Chinese version of Trauma Specific Frailty Index（C-TSFI）* | | | |
| --- | --- | --- | --- |
| **Comorbidities** | Cancer history | □Yes (1) □No (0) |  |
|  | Coronary Heart Disease | □Myocardial Infarction (1)  □Coronary Artery Bypass Grafting (0.75)  □Percutaneous Coronary Intervention (0.5)  □Medication (0.25)  □None (0) |  |
|  | Dementia | □Severe(1) □Moderate(0.5) □Mild(0.25) □No(0) |  |
| **Daily Activities** | Help with grooming | □Yes(1) □No(0) |  |
|  | Help managing money | □Yes(1) □No(0) |  |
|  | Help doing housework | □Yes(1) □No(0) |  |
|  | Help toileting | □Yes(1) □No(0) |  |
|  | Help walking | □Wheelchair(1)  □Walker(0.75)  □Cane(0.5)  □No(0) |  |
| **Health Attitude** | Feeling useless | □Most time(1) □Sometimes(0.5)  □Never(0) |  |
|  | Feel sad | □Most time(1) □Sometimes(0.5)  □Never(0) |  |
|  | Feel effort to do everything | □Most time(1) □Sometimes(0.5)  □Never(0) |  |
|  | Falls | □Most time(1) □Sometimes(0.5)  □Never(0) |  |
|  | Feel alone | □Most time(1) □Sometimes(0.5)  □Never(0) |  |
| **Function** | Intimate contact | □Yes(0) □No(1) |  |
| **Nutrition** | Albumin | □<3(1) □>3(0) |  |

We changed “Feel less useful” to “Feeling useless”, “Feel lonely” to “Feel alone” in Health Attitude, and “Sexual activie” to “Intimate contact” in Function.

*Appendix2 Fifteen Variable Trauma Specifc Frailty Index(TSFI)*

| **Item** | **Criterions** | | |
| --- | --- | --- | --- |
| **Comorbidities** |  |  |  |
| Cancer History | Yes (1) | No (0) |  |
| Coronary Heart Disease | MI (1) | CABG (0.75) | PCI (0.5) |
|  | Medication (0.25) | None (0) |  |
| Dementia | Severe (1) | Moderate (0.5) | Mild (0.25) |
|  | No (0) |  |  |
| **Daily Activities** |  |  |  |
| Help with grooming | Yes (1) | No (0) |  |
| Help managing money | Yes (1) | No (0) |  |
| Help doing household work | Yes (1) | No (0) |  |
| Help toileting | Yes (1) | No (0) |  |
| Help walking | Wheelchair (1) | Walker (0.75) | Cane (0.25) |
|  | No (0) |  |  |
| **Health Attitude** |  |  |  |
| Feel less useful | Most time (1) | Sometimes (0.5) | Never (0) |
| Feel sad | Most time (1) | Sometimes (0.5) | Never (0) |
| Feel effort to do everything | Most time (1) | Sometimes (0.5) | Never (0) |
| Falls | Most time (1) | Sometimes (0.5) | Never (0) |
| Feel lonely | Most time (1) | Sometimes (0.5) | Never (0) |
| **Function** |  |  |  |
| Sexual active | Yes (0) | No (1) |  |
| **Nutrition** |  |  |  |
| Albumin | <3(1) | >3(0) |  |

The TSFI score is average score of the 15 items, ranging from 0 to 1. The higher the score, the weaker the body
